# Multi‐Locus Nuclear Marker Assessment of Genetic Diversity in Swiss Orthoptera Unveils Conservation Status Limitations

**DOI:** 10.1111/mec.70392

**Published:** 2026-06-06

**Authors:** Inés Carrasquer Puyal, Julia Bilat, Camille Pitteloud, Christian Monnerat, Sofia Wyler, Jérémy Gauthier, Nadir Alvarez

**Affiliations:** ^1^ Natural History Museum of Geneva Geneva Switzerland; ^2^ Service des Forêts, de la Nature et du Paysage de L'etat du Valais Sion Switzerland; ^3^ Info Fauna Neuchâtel Switzerland; ^4^ Naturéum—State Museum of Natural Sciences Lausanne Switzerland; ^5^ Department of Ecology and Evolution, Biophore University of Lausanne Lausanne Switzerland

**Keywords:** genetic diversity, hybridisation capture, nuclear loci, orthoptera, systematic

## Abstract

With accelerating biodiversity loss, tracking both inter‐ and intraspecific diversity is critical, as within‐species variation underpins population viability and adaptive potential. We developed a new multi‐locus framework, and obtained an Orthoptera‐specific marker panel targeting 398 nuclear loci plus the mitogenome and ribosomal DNA to assess species boundaries, population structure and intraspecific diversity. We applied this approach to 645 specimens covering all 105 Swiss Orthoptera species, sampled during the nationwide Red List update. This multi‐locus dataset produced a well‐supported phylogeny and resolved several taxonomic ambiguities with direct conservation relevance. We demonstrate its ability to identify fine‐scale population structure and further illustrate its application to genetic diversity estimation. While species' IUCN threat levels were not correlated with genetic diversity, we found a significant negative association between genetic diversity and dependence on riparian habitats: river‐bank specialists showed lower diversity, likely reflecting the severe fragmentation and alteration of these ecosystems. Our results demonstrate that the application of new molecular tools provides valuable, complementary insights not only for taxonomy but also for conservation assessments. The development and application of genetic monitoring frameworks, such as the one presented here, are highly valuable for promoting the inclusion of genetic diversity in future conservation assessments.

## 1 Introduction

Biodiversity is typically evaluated at several hierarchical levels, all of which must be considered to gain a comprehensive understanding of the ongoing environmental crisis (Noss 1990). Ecosystem diversity refers to the variety of habitats, communities, and ecological processes; community diversity considers the interactions among coexisting species; species diversity accounts for species richness and abundance; and finally, genetic diversity refers to the variation of genes within populations, providing the raw material for adaptation and evolution (Frankham 2005). However, assessment and monitoring methodologies often focus on a single biodiversity level. This is the case for the International Union for Conservation of Nature (IUCN), which uses Red List Categories and Criteria to assess extinction risk at the species level, based on population size, decline rate, and geographic range (IUCN 2012). While this framework is essential for setting global conservation priorities, understanding biodiversity at other levels requires different tools. At the ecosystem or community level, ecologists use indices such as species richness to quantify diversity (Gotelli and Colwell 2001). The advent of molecular techniques, such as DNA barcoding that generally relies on a single organellar gene (e.g., the cytochrome c oxidase I (COI) gene in animal lineages (Hebert et al. 2003)), has revolutionised biodiversity monitoring by enabling rapid, large‐scale species identification based on curated reference libraries. However, for many clades, the reliance on a single organellar barcode lacks full resolution, owing to limited discriminatory power in recently diverged lineages, uniparental (usually maternal) inheritance, incomplete lineage sorting, and, in the animal case, the presence of nuclear mitochondrial pseudogenes (*numts*) that can confound identification (Dupuis et al. 2012). This is particularly problematic for risk assessments requiring accurate species delimitation and taxonomic resolution across entire taxonomic groups.

In recent years, the conservation research community has increasingly highlighted the significance of genetic biodiversity at the molecular level (Hoban et al. 2021). Indeed, within‐species genetic diversity underpins a population's resilience and its ability to adapt to environmental changes (Frankham 2005). While estimates suggest an overall 6% loss of genetic diversity since the Industrial Revolution (Leigh et al. 2019), a more recent calculation suggests that this figure could reach 10%–16% due to habitat destruction and land‐use change alone (Exposito‐Alonso et al. 2022). Such genetic erosion compromises adaptive capacity, reduces long‐term population viability, and increases vulnerability to future environmental stressors (Schwartz et al. 2007; Kardos et al. 2021). Crucially, even when census numbers rebound after a bottleneck, the genetic diversity lost can take hundreds to thousands of generations to recover through mutation alone, leaving populations genetically impoverished long after they appear demographically viable (Allendorf et al. 2010; Laikre et al. 2020; van Oosterhout et al. 2026). Species once numerically dominant have shown some of the steepest population declines in recent decades (van Klink et al. 2024). As their effective population size collapses, they are likely undergoing a silent erosion as a result. Although the IUCN Red List acknowledges the role of genetic exchange in population dynamics, it currently lacks standardised procedures for incorporating genetic data into threat evaluations (Forester et al. 2022; Garner et al. 2020). Integrating quantitative genetic metrics into future Red List assessments would therefore enable earlier detection of genetically vulnerable species and support more effective conservation strategies (Garner et al. 2020). Equally critical is uncovering spatial genetic structure within species since delimiting conservation units—specifically evolutionarily significant units (ESUs) and management units (MUs)—guides targeted conservation interventions (Coates et al. 2018; Moritz 1994; Palsbøll et al. 2007). Recognising these genetic subdivisions facilitates prioritisation of conservation efforts by focusing on units that contribute substantially to overall species genetic diversity and adaptive potential (Fraser and Bernatchez 2001).

Numerous types of genetic markers have been developed to unravel the intra‐specific component of biodiversity (O'Brien et al. 2022). These usually rely on a broad number of nuclear polymorphisms. Indeed, because single—generally organellar—barcodes are composed of only several hundred or at best a few thousand nucleotides, their limited length offers too little sequence variability to accurately evaluate intraspecific genetic variation, making them unsuitable for tracking genetic diversity across populations (Toews and Brelsford 2012). Previous studies using genome‐wide data (Jeon et al. 2024; Zoonomia Consortium 2020; Wang et al. 2026) and microsatellites (Spielman et al. 2004; Vitorino et al. 2019; Willoughby et al. 2015) have reported lower genetic diversity in threatened species compared to non‐threatened species in birds and mammals. However, this pattern is not consistent in all cases, as highlighted by Kuderna et al. (2023) and Schmidt et al. (2022), who found that genetic diversity did not always correlate with species' conservation status. Consequently, explicit inclusion of quantitative genetic metrics in IUCN Red List evaluations could highlight vulnerabilities in species that might otherwise be classified as non‐threatened under current criteria (Norderhaug et al. 2024). While access to such data and extensive sampling requirements have posed challenges, recent progress makes the integration of intra‐specific genetic monitoring both feasible and essential. Although whole‐genome sequencing provides the most comprehensive insight into genetic diversity, its large‐scale application remains impractical. Consequently, reduced‐complexity genomic approaches targeting multiple nuclear loci are increasingly recognised as suitable alternatives (Coissac et al. 2016). Multi‐locus methods offer a comprehensive view of both inter‐ and intraspecific genetic variation, able to improve species delimitation, and provide insights into population processes (Choi et al. 2019). These approaches enable the estimation of genetic diversity within individuals by measuring the proportion of heterozygous positions across multiple loci. As heterozygosity correlates negatively with inbreeding depression and deleterious mutations, and positively with adaptive potential, it is a powerful yet underused metric for informing IUCN threat levels (Kardos et al. 2021). Applied at broad taxonomic scales, this framework offers a time‐ and cost‐efficient alternative to population‐level estimates (Zoonomia Consortium 2020; Kuderna et al. 2023).

In this study, we develop and apply a multi‐locus marker set specific to Orthoptera, allowing both accurate taxonomic identification and an assessment of intraspecific diversity. Orthoptera is a well‐known order of insects frequently used as ecological indicators due to their sensitivity to environmental changes (Kenyeres et al. 2020). We explore the utility of Universal Single‐Copy Orthologs (USCO; (Simão et al. 2015)) and UltraConserved Elements (UCE; (Faircloth et al. 2012)) for both species identification and intraspecific diversity monitoring in Swiss Orthoptera. USCO and UCE represent highly‐conserved single‐copy regions, ideal to perform detailed phylogenomic analyses (Dietz et al. 2024; Zarza et al. 2018). More recently, these markers have proven effective for conducting demographic analysis, given the variable nature of their flanking regions (Stiller et al. 2021).

Orthoptera present significant taxonomic and conservation challenges that are not easily addressed through genetic approaches. A key issue is their large genome size (up to 21 Gb; Hawlitschek, Sadílek, et al. 2023), characterised by high rates of repeated elements, and the prevalence of mitochondrial pseudogenes and heteroplasmy (i.e., the co‐occurrence of multiple mitochondrial haplotypes within a single individual), which together hamper both species identification and population‐level inference. This complicates the differentiation between orthologous and paralogous sequences, increasing the risk of distorted downstream analyses (Hawlitschek, Bruns, et al. 2023). COI barcode sequencing has yielded species delineation patterns that often contradict classical taxonomy and fail to resolve intraspecific variation, particularly in complex groups like the Gomphocerinae subfamily (Hawlitschek et al. 2017; Nabholz 2024). These limitations must be considered more than purely technical inconveniences, as they impose constraints on the inferences that underpin conservation assessments. Moving beyond a single mitochondrial marker to a multi‐locus marker set circumvents the main pitfalls outlined above and provides recombining loci that carry population‐level variation. Such an approach is particularly timely in the context of Switzerland. According to the latest national Red List published by the Swiss Federal Office for the Environment (FOEN), 39% of the 105 well‐documented Orthoptera species present in Switzerland are considered as threatened (Monnerat et al. 2007). The upcoming Red List update offers a unique opportunity to ask whether IUCN threat categories, built on demographic and distributional criteria, align with the genetic diversity actually retained in populations. In addition, it enables the resolution of extant taxonomic questions. Furthermore, voucher specimens collected during Red List surveys are deposited in museum collections, where they constitute an underused but ideally suited source of material for genetic analyses. Building on these elements, this study investigates whether IUCN threat categories reflect actual levels of genetic diversity within Swiss Orthoptera.

More specifically, we aimed at:

1. Improving the performance of the previous barcoding approach in delineating species and reconstructing phylogenies by identifying a minimal set of nuclear markers, thus enabling a more scalable methodology applicable to practitioners.
2. Adapting the multi‐locus marker approach for application to museum specimens, broadening the potential for historical data integration into modern conservation strategies.
3. Performing estimates of intraspecific genetic diversity and examining the correlation between within‐species genetic diversity and IUCN conservation categories, together with ecological factors.
4. Investigating whether the multi‐locus marker set allows to identify spatial genetic structures at the within‐species level, thereby delineating conservation units that could inform targeted conservation efforts.

## 2 Material and Methods

### 2.1 Sampling and Library Preparation

Individuals were sampled during the update of the Swiss Orthoptera Red List between 2018 and 2022, yielding 645 specimens representing all 105 Orthoptera species reported in Switzerland, spanning both suborders (Ensifera and Caelifera) and seven families (Acrididae, Gryllidae, Gryllotalpidae, Rhaphidophoridae, Tetrigidae, Tettigoniidae, Tridactylidae). The individuals were pinned, dried and assigned to a GBIF barcode. Sampling followed a stratified design to capture intraspecific genetic variation, including: (1) at least one specimen per biogeographic area (FOEN 2021); (2) for species classified as threatened in the previous national Red List (CR, EN, VU, NT), three specimens per biogeographic area from different localities; (3) for species with unresolved taxonomic issues, multiple specimens from each species, subspecies, or form were considered (Dataset S1). In the case of species currently extinct in Switzerland but which were historically present (*Bryodemella tuberculata*, *Melanogryllus desertus*, *Paracinema tricolour*, *Xya variegata*, *Epacromius tergestinus*), one sample was obtained from the collections of the Natural History Museum of Geneva. The taxonomy established by Ivković et al. (2024) was used throughout the article.

We extracted DNA from a middle leg of each of the 645 samples using the Qiagen BioSprint 96 DNA Blood Kit on the BioSprint 96 workstation at the Department of Ecology & Evolution, University of Lausanne. Shotgun libraries were prepared following a modified version of the protocol used in (Suchan et al. 2016), based upon Tin and colleagues (Tin et al. 2014). Samples were identified using barcoded adaptors and indexed PCR primers allowing multiplexing of numerous samples.

### 2.2 Development of the Multi‐Locus Marker Set

Among the 200 well‐annotated mitochondrial genomes of Orthoptera families occurring in Switzerland available on NCBI, we selected eight mitogenomes (Table S2). The 13 mitochondrial genes were extracted and aligned to design non‐overlapping probes of 170 base pairs. At the time of the beginning of this study (October 2021), no complete ribosomal DNA (rDNA) had been published for Orthoptera on NCBI and only a few partial sequences were available, mainly for Caelifera. From the six reference genomes, only *Vandiemenella viatica* and *Xenocatantops brachycerus* contained the complete rDNA sequence. To fill this gap, we amplified and sequenced the ribosomal region of 10 Swiss orthopterans well distributed in the order phylogeny (Table S3) using universal eukaryote primers (Krehenwinkel et al. 2019). The amplification products were sequenced using a Flowcell MinION R9.4.1 on a MinIon device from Nanopore Technologies at the Geneva Natural History Museum. The newly produced sequences, the *V. viatica* and *X. brachycerus* sequences, were aligned to produce 170 bp non‐overlapping probes.

The Ultra Conserved Elements (UCEs) were identified using the six best orthoptera reference genomes available at the beginning of the study. The selected genomes ensured a balanced representation of both suborders Caelifera (Acrididae) and Ensifera (Gryllidae) families, namely *Gryllus bimaculatus* (GCA_017312745.1), *Laupala kohalensis* (GCA_002313205.1), *Locusta migratoria* (ik5GCA_000516895.1), *Teleogryllus occipitalis* (GCA_011170035.1), *Vandiemenella viatica* (GCA_019457785.1) and *Xenocatantops brachycerus* (GCA_900249655.1). Note that only draft genomes assembled into scaffolds were available. The genome of *Timema monikensis* was included as an outgroup. The repeated regions were hidden using RepeatMasker (Tarailo‐Graovac and Chen 2009). The PHYLUCE pipeline was used with default parameters to identify UCE loci and design probes to target them. In order to reduce the impact of low‐quality reference genomes and repeated regions, we performed an in silico capture on the genomes, successfully retaining 1843 loci (32%). Among them, we selected those UCE captured in at least six genomes and removed UCE with a length under 120 pb, and having more than 50% of variable sites or displaying more than 10% of divergence within Ensifera or Caelifera. Therefore, 290 UCE of 165, 7 bp on average were included in the multi‐locus marker set. The USCO have been identified by applying BUSCO v4 on the same genome set. Among the 1367 USCO from the insecta database (insecta_odb10) only those shared by at least 5 of the 6 Orthoptera species with an available genome were selected. To prevent exon‐intron discrepancies we designed the probes by extracting the first and last 120 base pairs from each USCO. The final set of selected USCO consisted of 54 USCO and 108 probes—given that the two 120 bp ends were generally separated by several 100 base pairs, we defined the region targeted by each of the 108 probes as an independent locus. The total number of orthologous nuclear loci targeted in the multi‐locus marker set is thus 398.

Probes targeting the ribosomal DNA, the mitogenome and the conserved nuclear regions were *de novo* synthesised separately at Twist BioScience. A primer (P1) was added to the 5′ end, while the T7 promoter was added at the 3′ end and to all probes for DNA amplification and transcription into RNA. We amplified each pool of DNA probes according to the manufacturer's guidelines and the product was transcribed into RNA with the HiScribe T7 High Yield RNA Synthesis Kit from New England Biolabs, incorporating Biotin‐16‐UTP.

### 2.3 Hybridisation Capture and Sequencing

The probes targeting the ribosomal DNA, the mitogenome and the 398 nuclear regions (UCE and USCO) were synthesised and converted into biotinylated RNA probes. Details on the design of the probes are provided in the Supporting Information. To account for variation in DNA quantity, quality, and marker representation across samples, we implemented an ‘equi‐read‐arity’ protocol (see Supporting Information, Figure S6). The final product of the captures was pooled at a ratio of 9:1 for UCE + USCO and mitogenome + ribosomal DNA, respectively, and sequenced on Illumina NextSeq with 150‐bp reads (Fasteris, Geneva, Switzerland).

### 2.4 Bioinformatic Analyses

Raw reads demultiplexing and cleaning were performed using Cutadapt v2.0 (Martin 2011) to remove adapters, low‐quality bases (Phred < 10), reads shorter than 30 bp, and unpaired reads. Read quality was checked using FastQC (https://www.bioinformatics.babraham.ac.uk/projects/fastqc/). Reads were assembled, and the PHYLUCE pipeline (Tarailo‐Graovac and Chen 2009) was used to directly map probes and identify orthologs. However, assembly was limited by genome gigantism, and ortholog recovery was affected by paralogy. During the course of this study, high‐quality reference genomes for *Meconema thalassinum* , *Schistocerca gregaria*, *Eucriotettix oculatus*, and *Gryllus longicercus* became available and were used as family‐specific or closely related references for read mapping with BWA‐MEM (Table S1). Alignments were subsequently filtered (MAPQ ≥ 20), and consensus sequences were generated using samtools (Li et al. 2009) and bcftools (Danecek et al. 2021). Identification of the UCE and USCO orthologous regions was performed on the reference genomes, combining the PHYLUCE pipeline (Faircloth 2016), BUSCO v4 (Seppey et al. 2019), and mapping of the probes with BWA‐MEM (Li 2013) (Figure S1). Additionally, we individually aligned the UCE and USCO probes with each region of the reference genomes where at least three samples mapped using MAFFT v7.407 (Katoh and Standley 2013) to obtain a distance value using distmat from EMBOSS v6.6.0.0 (Rice et al. 2000). Regions with a distance value below 30 were classified as corresponding UCE or USCOs(Figure S2). Genomic context was determined using available annotations or gene prediction with augustus (Hoff and Stanke 2019). Ribosomal loci and mitogenomes were assembled using SPAdes (Bankevich et al. 2012). Ribosomal loci were reconstructed using the RagTag tool (Alonge et al. 2022) based on BLAST Reciprocal Best Hit (RBH) (Camacho et al. 2009), while mitochondrial genes were identified using BLAST and complemented with MitoFinder (Allio et al. 2020). COI barcode regions were extracted by BLASTing full‐length COI sequences against family‐specific BOLD databases (Ratnasingham et al. 2024) (Figure S1). To account for paralogy and missing data, loci were aligned with MAFFT v7.407 (Katoh and Standley 2013), trimmed with trimAL v1.4.rev15 (Capella‐Gutiérrez et al. 2009) and sequences with > 40% of missing values were removed. Paralogs were filtered using FilterParalog.py from ORTHOSKIM v 1.6 (Pouchon et al. 2022) in three consecutive rounds at the order, family and genus levels.

### 2.5 Phylogenetic Inferences

Phylogenetic inferences were performed separately for (i) nuclear UCE and USCO, (ii) ribosomal DNA, (iii) full mitochondrial DNA and (iv) mitochondrial COI barcode alone. We considered each of the families with more than one species occurring in Switzerland separately (Acrididae, Gryllidae, Rhaphidophoridae, Tetrigidae, Tettigoniidae). Loci shared by at least one‐third of the samples and samples with less than 20% of missing data were kept for phylogenetic analysis. After concatenation, we used PartitionFinder 2.1.1 (Lanfear et al. 2017) to estimate the best partitioning schemes and ModelFinder (Kalyaanamoorthy et al. 2017) implemented in IQ‐TREE 2.0.5 (Minh et al. 2020) to select corresponding models of nucleotide substitution. To avoid local optima, we performed 25 independent tree searches for each data set in IQ‐TREE 2.0.5 using default settings. Branch support was estimated by calculating 1000 ultrafast bootstraps replicates and 1000 SH‐aLRT tests in IQ‐TREE 2.0.5 with tree optimisation by NNI on bootstrap alignment.

### 2.6 Genetic Diversity Estimations

To identify the percentage of variant sites within individuals, we used the nuclear loci considered for the phylogenetic inference. Samples' reads were mapped to their own corresponding UCE and USCO loci, ribosomal DNA, mitogenome and COI barcode using BWA‐MEM (Li 2013). SNP calling was performed using GATK v4.1.2.0 (McKenna et al. 2010), retaining only biallelic SNPs with a minimum coverage of six reads and a minimum quality score of 100. We retained only exonic regions due to their higher proportion compared to intronic regions and their constraining evolutionary rates. Genetic diversity was calculated per individual by dividing the number of variant sites found in exonic regions by the total exon length covered by at least six reads (i.e., a measure of the fraction of polymorphic sites computed as the percentage of heterozygous positions within individuals). Then, the mean value per species was calculated. This measure of *alpha* genetic diversity, corresponds to the first level of the Essential Biodiversity Variables (EBVs) recently developed by the Group on Earth Observations Biodiversity Observation Network (GEO BON) (Hoban et al. 2022). Percentages of polymorphic sites obtained from the nuclear loci were compared to those from the ribosomal DNA, the mitogenome, and the COI barcode using a regression model (R package stats). Only the values from the nuclear loci were retained for further analysis.

To investigate putative links between species' overall genetic diversity and ecological factors as well as IUCN category, we used the complete Swiss database of species' ecological preferences and biological properties, Fauna Indicativa (Klaiber et al. 2017), along with the level of threat assigned during the 2007 Red List assessment (Monnerat et al. 2007). After removing correlated variables using cor (R package stats), the final list of variables was: IUCN categories, habitat specialisation, riparian indicator species, dry meadows and pastures indicator species, dispersal potential, thermophilic preference, elevation level and percentage of polymorphic sites. We investigated associations using PCA (R package ade4) and a generalised linear model (R package stats). The difference between threatened (VU + EN + CR) and non‐threatened (LC + NT) species was tested using a two‐sided Wilcoxon test. Museum specimens were excluded from this analysis because they belonged to the time period prior to the Red List assessment.

### 2.7 Intraspecific Biogeographic Structure

To examine whether the UCE/USCO multi‐locus marker set was able to detect within‐species genetic variation and retrieve species' spatial genetic structure, we selected within the dataset the four species with more than 15 samples, *Psophus stridulus*, *Oedipoda caerulescens*, *O. germanica* and *Stethophyma grossum*. We retrieved UCE and USCO mappings regions used to generate the phylogenies and conducted SNP calling using freeBayes v1.3.2 (Garrison and Marth 2012). Biallelic neutral SNPs with a minimum quality score of 100 and a maximum missing rate of 0.85% at both SNP and individual levels were selected using vcftools (Danecek et al. 2011). We used this SNP dataset to perform a PCA (R package ade4), conduct k‐means clustering (R package stats) and determine the optimal number of k‐means clusters based on the highest average silhouette width (R package factoextra).

## 3 Results

### 3.1 Capture Efficiency

Following demultiplexing of the 645 specimens, we obtained a total of 250 million reads, averaging 370,929 reads per sample (SD = 301,478). The percentage of mapped reads varied across families (Table S1). The orthology inference step performed on nuclear loci identified 186 UCE and USCO loci in *Meconema thalassinum* , 339 in *Gryllus longicercus*, 321 in *Schistocerca gregaria*, and 249 in *Eucriotettix oculatus*. The USCO probes were, by definition, placed at the end of exons. 56% of UCE were located at the exon‐intron junctions, 44% were entirely exonic but none was placed at the intergenic regions. Overall, 93% of captured sequences were exonic. Their exact location within each genome is provided in Supporting Information. The number of UCE and USCO captured differed across families (Figure S3). The capture strategy enabled inclusion of 449 samples for phylogenetic analyses and 405 for genetic diversity analyses, with an average recovery of ~140 loci per sample. Whereas we initially aimed to develop a single multi‐locus marker set for all Orthoptera species, this proved impractical due to the ancient divergence among infraorders and the limited number of shared genetic loci across them. To address this, we instead defined four sets of multi‐locus marker sets, each tailored to the genetic characteristics of a particular family (see Supporting Information for sample metadata and capture summary statistics).

### 3.2 Phylogenetic Inference

**FIGURE 1:**
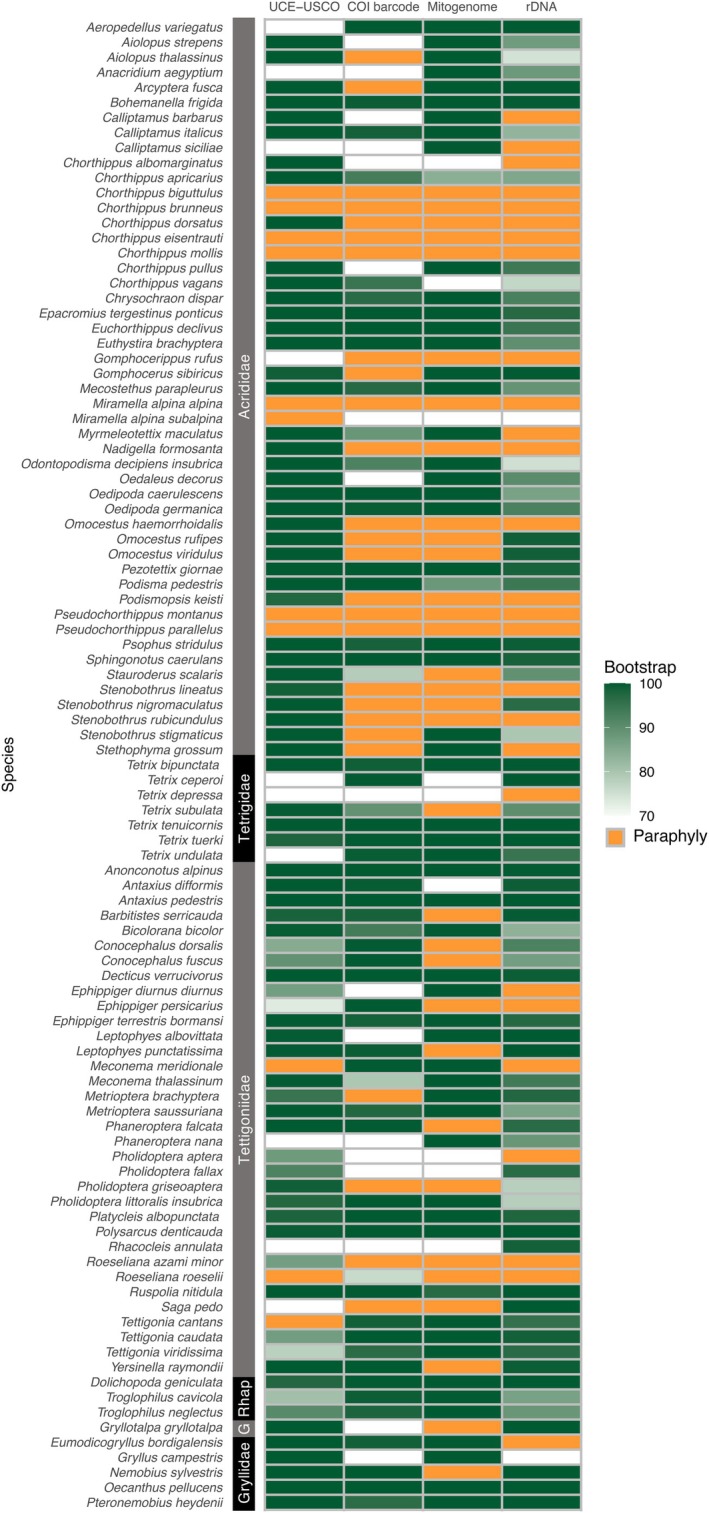
Comparison of the node support for each Orthoptera species occurring in Switzerland according to different markers. Colour scale corresponds to the value of bootstrap, while orange indicates paraphyly. UCE‐USCO stands for UltraConserved Elements and Unique Single Copy Orthologs, and rDNA for ribosomal DNA. Only species with at least 2 samples were included. Rhap stands for Rhaphidophoridae and G for Gryllotalpidae.

To investigate the species discrimination power of our set of loci, phylogenetic inferences were performed using a maximum likelihood approach. Using nuclear loci we obtained a greater overall support for the monophyly of species, accurately identifying 78 of 99 species as monophyletic (Figure 1), compared to single‐locus and plasmid barcodes (i.e., ribosomal DNA, mitogenome and COI). When limiting the comparison to the 77 species that were sufficiently sampled across all markers, UCE and USCOs outperformed the other markers, identifying 67 species as monophyletic. In contrast, 57 species were identified as monophyletic with rDNA, 53 with the COI barcode, and 46 with the entire mitogenome. Additionally, UCE and USCO loci displayed more robust results in resolving deeper evolutionary relationships, with higher supports and longer branch lengths. Including additional mitochondrial genes alongside the COI barcode did not increase the number of monophyletic species identified; in fact, it reduced the total number of identifiable species and produced less accurate topologies compared to the COI barcode alone. Ribosomal DNA sequences improved the monophyletic species yield compared to mitochondrial regions. However, polytomies frequently occurred at the intraspecific level, along with overall short branches and low node support, reducing its informativeness.

Given the limitations of the complete mitogenome and ribosomal DNA, we focused on the comparison between phylogenies obtained from the COI barcode, which has been widely used for species identification over the last two decades, and those generated using UCE and USCO loci (Figure 2, Figures S7–S16).

**FIGURE 2:**
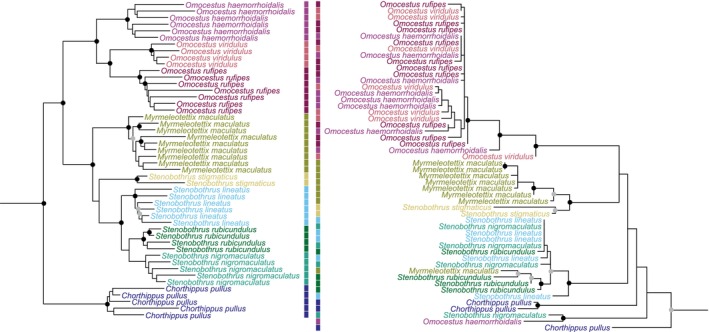
Phylogenetic reconstruction for the Stenobothrini clade using 260 UltraConserved Elements (UCE) and Unique Single Copy Orthologs (USCO) (left) and the COI barcode (right), for the sake of illustrating the better performance of the UCE + USCO marker set compared to the use of the COI barcode alone. Black nodes: UFBoot > 95 and SH‐aLRT > 80; grey nodes: either support.

Within the Acrididae family, the phylogenetic tree constructed using nuclear loci significantly differs from the tree inferred from the COI barcode and demonstrates better resolution, identifying 37 of 49 species as monophyletic versus 19 species only with COI. When considering only the species represented in both phylogenies, the numbers of monophyletic species retrieved are 32 of 39 for nuclear loci versus 18 with COI. The multi‐locus marker set recovers all Swiss species of *Omocestus* and *Stenobothrus* as monophyletic and places *Stenobothrus* as a coherent genus with *Myrmeleotettix maculatus* correctly positioned as an outgroup. Instead, the COI barcode fails to resolve species boundaries within these genera and retrieves *Stenobothrus* as paraphyletic. In both phylogenies, *M. maculatus* and *Chorthippus pullus*, although members of the Gomphocerini tribe, are nested within the Stenobothrini clade (Figure 2). *Nadigella formosanta* is nested within *Miramella alpina*, which itself splits into two geographic clades under nuclear loci but not with COI data. However, the *Chorthippus biguttulus* complex remains unresolved across all markers and within the Melanoplini tribe. For the Tettigoniidae family, despite the limited number of UCE and USCO loci captured, the family's phylogeny achieved good species‐level resolution. Using 27 UCE and USCO markers, 28 of 34 species were retrieved monophyletic, four more than with the COI barcode alone. However, in absolute terms, when considering only the 27 species successfully captured by both markers, the total number of species identified by each method is identical, that is, 24. For instance, both COI and multi‐locus marker set phylogenies recovered *Leptophyes* and the Platycleidini tribe as paraphyletic while supporting the monophyly of the genus *Metrioptera*. Regarding the phylogenies of Tetrigidae, Rhaphidophoridae and Gryllidae families, 96, 29 and 79 nuclear loci were retained, respectively. All occurring species were successfully retrieved as monophyletic using the multi‐locus marker set and COI. In the case of Tetrigidae, the two subspecies of *Tetrix bipunctata*, ssp. *bipunctata* and ssp. *kraussi* did not form separate clades. Both multi‐locus marker set and COI barcode phylogenies as well as their interpretation and discussion can be found in Supporting Information. Seven of the 16 museum specimens included in the study could be included in the phylogenetic analysis. They allowed investigating the placement of *Podismopsis keisti*, the only endemic species at the Swiss scale, with respect to its sister species *Podismopsis transsylvanica*, sampled in the collection of the Natural History Museum of Geneva. Despite the reduced sampling, *P. transsylvanica* is consistently placed as an outgroup of the *P. keisti* samples, reinforcing the conservation status of both rare species.

### 3.3 Genetic Diversity Estimation

**FIGURE 3:**
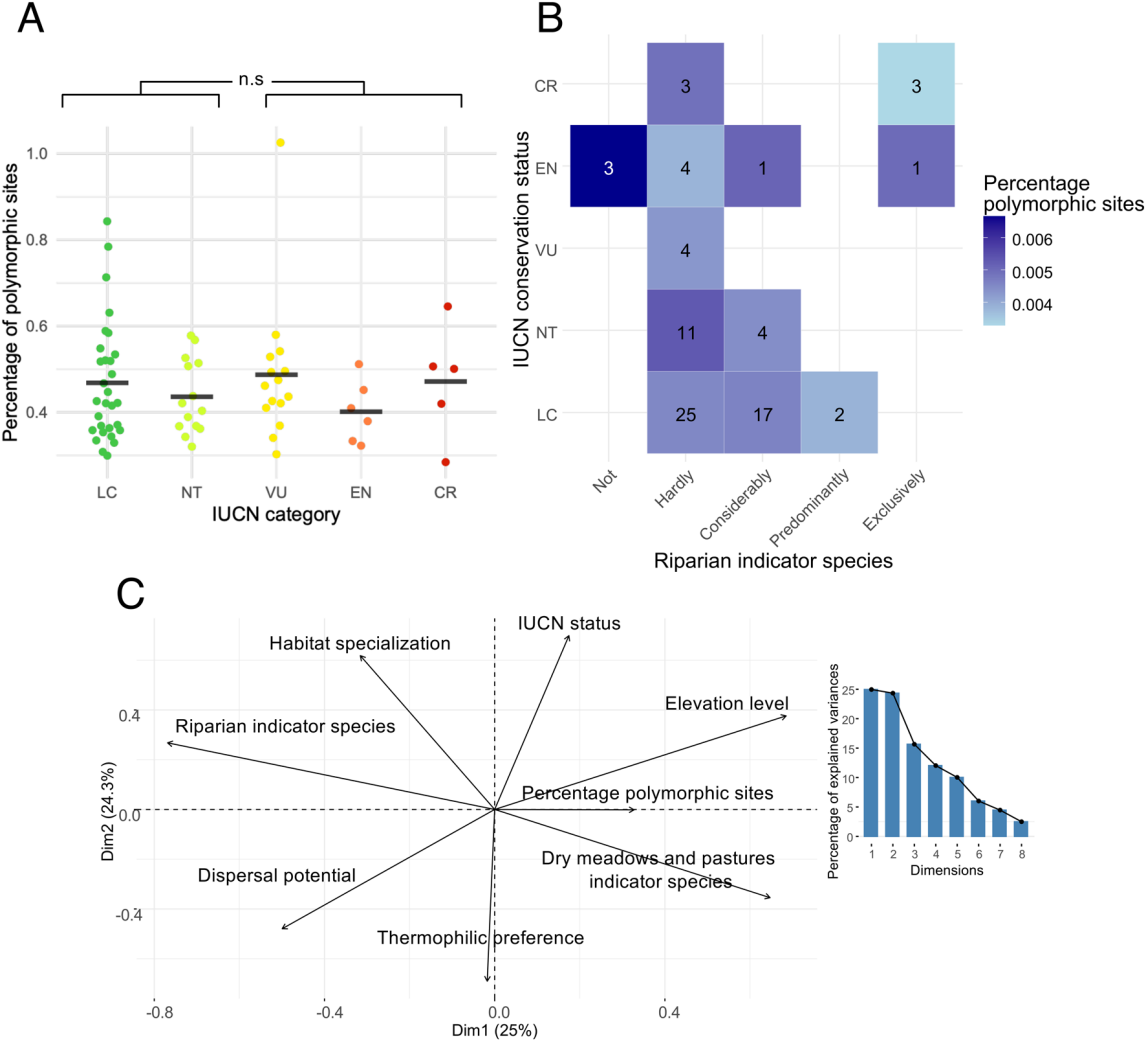
Distribution of genetic diversity in Swiss Orthoptera related to ecological and conservation variables. (A) Mean percentage of polymorphic sites per IUCN conservation category. The non‐significant *p*‐value for the two‐sided wilcoxon test is indicated on the top. CR, critically endangered; EN, endangered; LC, least concern; NT, near threatened; VU, vulnerable. (B) Distribution of the percentage of polymorphic sites according to the interaction between IUCN conservation categories and the degree of species’ riparian habitat association. The numbers within the tiles represent the number of species in each category. (C) Principal Component Analysis plot showing variables derived from Fauna Indicativa (Klaiber et al. 2017) and the mean percentage of polymorphic sites per species. The percentage of polymorphic sites was calculated as the proportion of heterozygous positions within Ultra‐Conserved Elements (UCE) and Unique Single Copy Orthologs (USCO) loci for each sample. The eigenvalues for each principal component are represented on the right panel.

Despite the presence of variable sites in ribosomal DNA and the mitogenome (including COI), none of these markers showed a correlation with the diversity observed in the nuclear loci (Figure S4). The percentage of polymorphic sites per species did not show a relationship with the geographical area occupied by the samples (Figure S5). The Principal Component Analysis (Figure 3C) indicated that species with a higher percentage of polymorphic sites tend to be those inhabiting higher elevations and dry meadow and pastures species, for example, *Aeropedellus variegatus*, *Ephippiger terrestris bormansi* or *Arcyptera fusca*. In contrast, riparian species, habitat specialists and species with high dispersal potential tend to display lower diversity levels, for example, *Chorthippus pullus*, *Epacromius tergestinus ponticus* or *Pteronemobius lineolatus*. A generalised linear model (Table 1) indicates that the riparian indicator status was the only factor demonstrating a significant correlation with the percentage of polymorphic sites, whereas neither habitat preferences nor the IUCN conservation category showed a significant correlation with the within‐species genetic diversity (Table 1). Genetic diversity did not differ between threatened and non‐threatened species (Wilcoxon rank sum exact test: *p*‐value = 0.899; Figure 3A). When the mean percentage of polymorphic sites exhibited by each species was compared to their riparian indicator and IUCN conservation category, we found two species, *Aiolopus thalassinus* and *Pteronemobius heydenii*, which are classified as Least Concern despite being predominantly floodplain species and exhibiting low levels of genetic diversity (Figure 3B).

**TABLE 1:** Results of the Generalised Linear Model (GLM) analysing the relationship between the mean percentage of polymorphic sites per species and the ecological variables extracted from Fauna Indicativa (Klaiber et al. 2017).

	Estimate	Std. error	*t*‐value	Pr(>|*t*|)
Intercept	−5.40558	0.31238	−17.305	< 2e−16***
IUCN conservation category	0.01083	0.03137	0.345	0.7311
Habitat specialisation	0.01068	0.06324	0.169	0.8664
Elevation level	0.06904	0.05297	1.304	0.1971
Riparian indicator species	−0.11298	0.05206	−2.170	0.0338*
Dry meadows and pastures indicator species	−0.05065	0.03840	−1.319	0.1919
Dispersal potential	0.01590	0.04879	0.326	0.7456
Thermophilic preference	0.07738	0.06900	1.121	0.2663

*Note:* The percentage of polymorphic sites was obtained as the percentage of heterozygous positions within individuals in the Ultra‐Conserved Elements (UCE) and Unique Single Copy Orthologs (USCO) captured. Significance codes: ***p < 0.001, **p < 0.01, *p < 0.05, *p* < 0.1; none = *p* ≥ 0.1.

### 3.4 Intraspecific Biogeographic Structure

**FIGURE 4:**
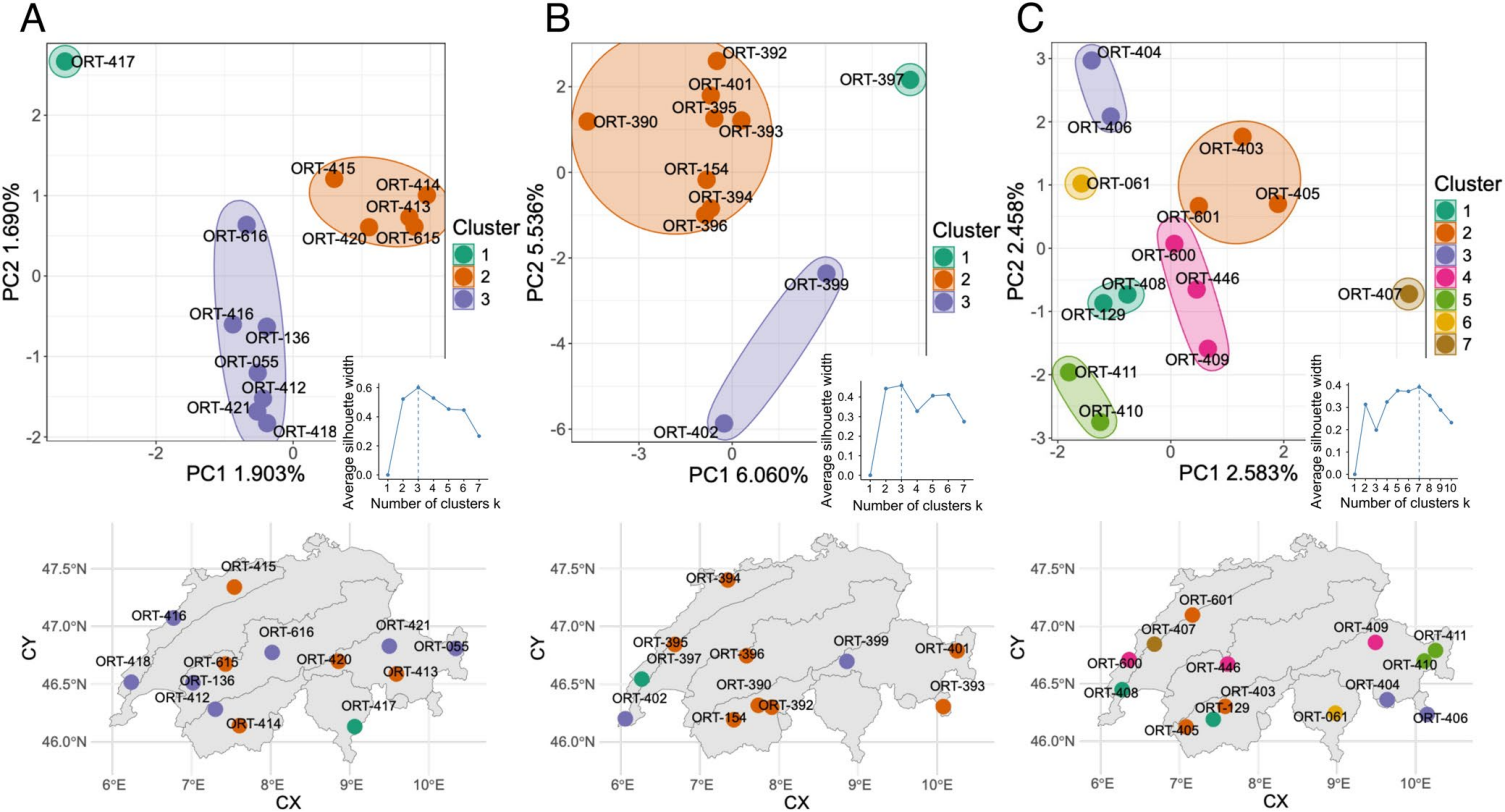
Spatial genetic structure of *Psophus stridulus* (A), *Oedipoda caerulescens* (B) and *Oedipoda germanica* (C) in Switzerland. The structuring is derived from Single Nucleotide Polymorphisms (SNPs) identified within Ultra‐Conserved Elements (UCE) and Unique Single Copy Orthologs (USCO) in each species followed by a k‐means analysis. The maps of Switzerland display the six biogeographical regions, illustrating the distribution of clusters across these regions.

For the four species sampled with ≥ 15 individuals, that is, *P. stridulus* , *O. caerulescens* , *O. germanica* and *S. grossum*, we investigated spatial genetic structure at the country‐wide level. After SNP filtering, we retained 586, 546 and 230 SNPs for *P. stridulus* , *O. caerulescens* and *O. germanica* , respectively. No SNP passed the filters for *S. grossum*, therefore no spatial genetic structure analysis could be performed for this species. For *P. stridulus* , the optimal number of k‐means clusters was three, based on the highest average silhouette width. Cluster 1, which is the most differentiated, was located south of the Alps, in the canton of Ticino (Figure 4A). Clusters 2 and 3 displayed a mixed distribution across the rest of Switzerland, suggesting some genetic structuring but with gene flow or shared genetic ancestry across these regions. For *O. caerulescens* , three clusters were identified; however, their geographical distribution suggests a complex geographical scenario at the Swiss level (Figure 4B). In *O. germanica* , a more detailed structured pattern with seven clusters was retrieved, indicating finer genetic distinction among samples. Some clusters demonstrated a localised geographical distribution, such as in the east (in green in Figure 4C) or in the south (in purple and yellow in Figure 4C). For the other clusters, the geographical pattern is less pronounced.

## 4 Discussion

As the sixth mass extinction unfolds (Ceballos et al. 2015), efficient tools are urgently needed to monitor biodiversity at both inter‐ and intra‐specific scales. We evaluated a set of nuclear loci (UCE and USCO) as a multi‐locus barcoding system for species identification, genetic diversity estimation, and spatial structure inference, and benchmarked its performance against the informativeness of the rDNA cluster, the complete mitogenome, and the COI barcode in particular. We applied this framework to Orthoptera, an insect order of ecological and conservation concern (Dvořák et al. 2022) with large and complex genomes (Hawlitschek, Sadílek, et al. 2023) during the update of the Swiss Red List.

### 4.1 Multi‐Locus Marker Efficiency

Although we initially aimed to identify universal loci across Orthoptera, the deep evolutionary history and strong divergence among clades needed the development of four family‐specific locus sets. Similar strategies have recently been adopted in other insect groups, such as Adephaga (Gustafson et al. 2020) or the Noteridae (Baca et al. 2024). Unlike other existing Orthoptera probe sets designed to resolve deep phylogenetic nodes (Shin et al. 2024), we deliberately limited the number of loci to optimise the trade‐off between marker resolution and sample inclusion, facilitating scalable and reproducible applications in conservation contexts.

### 4.2 Phylogenetic Inference

Overall, we observed an improvement in the phylogenetic resolution for Orthoptera using UCE and USCO markers compared to COI barcode, the full mitogenome, or the ribosomal DNA, individually. Phylogenetic trees inferred from the multi‐locus marker sets provided robust support across both deep and shallow evolutionary nodes and resolved many discrepancies between traditional taxonomic classifications and barcoding‐based identifications of individuals, as noted by Hawlitschek et al. (2017). In contrast, the COI, full mitogenome and ribosomal based inference performed more poorly in resolving species as well as taxonomic relationships beyond the species level, failing to capture the full complexity of genetic relationships. Note that for most species, only COI barcode phylogenies were previously available (Hawlitschek et al. 2017).

In Acrididae, previous phylogenomic studies have primarily focused on the radiation of subfamily Gomphocerinae (Schmidt et al. 2024) or Melanoplinae (Chintauan‐Marquier et al. 2014). We provide a well‐supported phylogenetic reconstruction of Acrididae, confirming the monophyly of most species and resolving several taxonomic ambiguities. For the Tetrigidae species considered, only COI barcode phylogenies were available (Hawlitschek et al. 2017), and their topology differed markedly from that obtained using nuclear loci, illustrating a case of mito‐nuclear discordance. Research on Tettigoniidae (katydids) has relied on ribosomal DNA and mitochondrial genes, with limited genomic studies available that typically include only a few species occurring in Switzerland (Shin et al. 2024), which makes comparison difficult. However, we inferred a well‐supported and resolutive phylogeny, outperforming the one obtained with COI barcode. Finally, the results provide genome‐level data for most of the Gryllidae, Gryllotalpidae, and Rhaphidophoridae species, as prior studies have primarily used mitochondrial or ribosomal DNA. These phylogenetic results directly informed the Red List assessment by supporting taxonomic revisions and clarifying species and subspecies boundaries.

### 4.3 Genetic Diversity Estimation

The use of a multi‐locus marker set to identify intra‐individual genetic variation, that is, individual‐based heterozygosity, has shown to be a relevant method to investigate intraspecific genetic diversity (Petersen et al. 2022; Samuels et al. 2016). This is particularly important for species‐rich groups, where population‐level sampling would make broad‐scale assessments logistically and financially unfeasible. The discrepancy between ribosomal DNA, mitochondrial, and nuclear diversity may reflect maternal inheritance, heteroplasmy and numts, or intragenomic variation in ribosomal DNA copies, which are well documented in Orthoptera (Keller et al. 2006). The diversity estimates provided should be interpreted as a standardised measure across individuals and species, providing comparable information representative of genome‐wide diversity levels. Despite the loci being located in exonic regions, the variants were mostly found in the flanking regions. It is difficult to consider them neutral without knowing the selection pressures that apply to the genes, but they do not necessarily represent adaptive diversity either.

Links between genetic diversity and extinction risk have remained largely theoretical and are not incorporated into Red Lists. Previous studies, mostly in mammals, reported higher genetic diversity in Least Concern and Near Threatened species than in threatened taxa (Jeon et al. 2024; Zoonomia Consortium 2020). In contrast, we found no association between IUCN status and intraspecific genetic diversity in Swiss Orthoptera. Among the factors that could explain this decorrelation is the fact that in species having rebounded in census size after a severe bottleneck, populations can remain genetically impoverished for a large number of generations (Lucena‐Perez et al. 2021) and, conversely, in species with declining populations, the loss of genetic diversity may be delayed or not immediately apparent (Gargiulo et al. 2025). In either case, the loss of genetic diversity may trigger a self‐reinforcing process known as the extinction vortex (Forester et al. 2022).

### 4.4 Limited Drivers of Within‐Species Genetic Diversity: The Case for Riparian Species

To explore further the link between heterozygosity levels and other variables, and owing to the ecological data available for the species, we investigated the factors correlating with the estimates of genetic diversity. Whereas a trend towards a negative association between within‐species heterozygosity and both elevation level and degree of indicator species of dry meadows and pastures was found in a multivariate analysis, we identified only one significant correlation between diversity metrics and one ecological species trait, that is, the degree of riparian species status. We could identify species predominantly related to riparian habitats and currently associated to Low Concern IUCN status, for example, *Aiolopus thalassinus* and *Pteronemobius heydenii*, which may warrant higher conservation priority due to their low levels of heterozygosity. Other species, exclusively associated with riparian habitats, namely *Chorthippus pullus*, *Epacromius tergestinus ponticus* and *Tetrix tuerki*, are already classified as Critically Endangered. The correlation between genetic diversity and riparian species status mirrors the ~90% loss of Swiss floodplains since 1850 (Müller‐Wenk et al. 2004). With the exception of the riparian species status, we cannot thus use an ecological factor as a proxy for a species' genetic diversity. This indicates that neither thermophilous nor alpine species exhibit higher genetic diversity than species more impacted by climate change or habitat disturbance. This result is in line with results shown by De Kort et al. (2021), who demonstrated that genetic diversity does not systematically rise with temperature or elevation across global plant and animal populations. Results overall call for revising the IUCN categories of all orthopteran species, by including genetic diversity metrics. Such integration is essential, as our findings show that neither ecological traits nor current Red List status reliably capture genetic erosion.

### 4.5 Intraspecific Biogeographic Structure

Beyond reconstructing species phylogenetic relationships at the family level, the multi‐locus marker also enabled the determination of intra‐specific genetic structuring. Population analysis across three of the four most sampled Orthoptera species in the dataset (all three Near Threatened according to the IUCN) revealed population differentiation. In *P. stridulus* and *O. germanica* , the results evidence distinct geographical clustering, which could be considered as several conservation units of interest. Whereas UCE are increasingly used to estimate within‐species genetic structure, most studies use a large number of UCE over broad geographic areas (Azevedo et al. 2024; Nikolakis et al. 2021). Overall, these results demonstrate the power of the UCE and USCO multi‐locus marker sets established here to uncover fine‐scale genetic structure relevant for defining conservation units, even when using a limited number of loci.

### 4.6 Minimal Multi‐Locus Marker for Monitoring Strategy

We evaluated the performance of UCE and USCO for estimating within‐species genetic diversity and detecting intraspecific genetic structure at a country‐wide scale in an insect order. This study demonstrates the practical applicability of reduced‐complexity genomic approaches, helping to bridge the conservation genetics research–implementation gap (Hogg 2024; Taylor et al. 2017). Using a limited number of informative loci distributed across the genome optimises the trade‐off between marker resolution and sample size, making this strategy particularly suitable for large‐scale monitoring across entire taxonomic groups. The development of molecular tools relying on a streamlined set of orthologous loci, informative not only across both shallow and deep phylogenetic scales but also at the population level, enhances species delimitation capabilities while enabling detection of intraspecific genetic diversity and spatial genetic structuring relevant for conservation planning.

## Author Contributions

N.A., I.C.P., C.P., J.G., and S.W. conceived and designed the study. C.M. and S.W. coordinated sample collection. I.C.P. and J.B. conducted the laboratory work. I.C.P., J.G., and N.A. performed the bioinformatic analyses and interpreted the results. I.C.P. wrote the manuscript with revisions from J.G. and N.A. All authors revised the manuscript and approved the final version. J.G. and N.A. contributed equally to this work as senior authors.

## Funding

This work was supported by Federal Office for the Environment FOEN (05.0039.PZ/S154‐1406).

## Disclosure

All scientific information generated by this project, including biological inventories, taxonomic insights, and genetic diversity data, is made openly accessible and shared with conservation authorities to support biodiversity assessment and sustainable management. Research results, methodologies, and analytical tools developed using genetic resources are shared with providers under fair and favourable terms, contributing directly to conservation practice and capacity building. The project further promotes training and knowledge exchange in molecular and conservation genomics.

## Conflicts of Interest

The authors declare no conflicts of interest.

## Supporting information


**Figure S1:** Bioinformatic pipeline used to retrieve UltraConserved Elements (UCE), Unique Single Copy Orthologs (USCO), ribosomal DNA, mitogenome and COI barcode from raw data. For UCE and USCO, orthology inference was conducted on four reference genomes using (i) PHYLUCE, (ii) BUSCO v4, (iii) probe mapping and (iv) alignment distance thresholds. Ribosomal DNA and mitochondrial genomes were reconstructed using SPAdes, BLAST as well as RagTag and MitoFinder, respectively. COI barcodes were extracted via BLAST using BOLD database references.
**Figure S2:** Distribution of genetic distances between captured genomic regions for each reference genome and the probes used to target the UltraConserved Elements (UCE) and Unique Single Copy Orthologs (USCO). The correct group corresponds to distances between the probes and loci previously identified as UCE or USCO through mapping or using PHYLUCE pipeline or BUSCO v4. The dotted line indicates the maximum distance accepted for a region to be assigned as a UCE or USCO.
**Figure S3:** Capture success of the multi‐locus marker. A: Heatmap of UltraConserved Elements, Unique Single Copy Orthologs, ribosomal DNA, mitochondrial genes and COI barcode. B: Distribution of the number of UltraConserved Elements and Unique Single Copy Orthologs capture per sample according to their family. Numbers in the upper squares indicate the number of samples per category.
**Figure S4:** Correlation between the mean % of polymorphic sites found in the UltraConserved Elements and Unique Single Copy Orthologs and the % of polymorphic sites found in the COI barcode (A), the mitogenome (B) and the ribosomal DNA (C). The blue line indicates linear regression.
**Figure S5:** Correlation between the mean % of polymorphic sites found in the UltraConserved Elements and Unique Single Copy Orthologs per species and the area (km^2^) occupied by the samples. The blue line indicates linear regression.
**Figure S6:** A. Schematic representation of the “equi‐read‐arity” process and its integration into final capture. B. Coverage plots and pooling categories.
**Figure S7:**. Phylogeny of the Acrididae family inferred using a maximum likelihood approach based on 260 Ultra‐Conserved Elements and Unique Single‐Copy Orthologs. Arcy. stands for Arcypterini, C. for Cyrtacanthacridini Chry. for Chrysochraontini, Epac. for Epacromiini, Gomp. for Gomphocerini, Locu. for Locustini, P. for Pezotettiginae and Sphi. for Sphingonotini. Black nodes indicate UFBoot ≥ 95 and SH‐aLRT ≥ 80; grey nodes indicate either UFBoot ≥ 95 or SH‐aLRT ≥ 80.
**Figure S8:**. Phylogeny of the Acrididae family inferred using a maximum likelihood approach based on the COI barcode. A. stands for Arcypterini, B. for Bryodemini, C. for Cyrtacanthacridini, Call. for Calliptamini, E. for Epacromiini, G. and Gomp. for Gomphocerini, L. for Locustini, P. for Pezotettiginae, Para. for Parapleurini, Podi. for Podismini, S. and Sten. for Stenobothrini and Sphi. for Sphingonotini. Black nodes indicate UFBoot ≥ 95 and SH‐aLRT ≥ 80; grey nodes indicate either UFBoot ≥ 95 or SH‐aLRT ≥ 80.
**Figure S9:**. Phylogeny of the Tettigoniidae family inferred using a maximum likelihood approach based on 27 Ultra‐Conserved Elements and Unique Single‐Copy Orthologs. Barb. and B. stand for Barbitistini, Copi. for Copiphorini, Cono. for Conocephalini, Ephi. for Ephippigerini, M. for Meconematini, P. for Platycleidini, Phan. for Phaneropterini and S. for Saginae. Black nodes indicate UFBoot ≥ 95 and SH‐aLRT ≥ 80; grey nodes indicate either UFBoot ≥ 95 or SH‐aLRT ≥ 80.
**Figure S10:**. Phylogeny of the Tettigoniidae family inferred using a maximum likelihood approach based on the COI barcode. Copi. stands for Copiphorini, Cono. for Conocephalini, Ephi. for Ephippigerini, Meco. for Meconematini, P. for Platycleidini, Phan. for Phaneropterini and S. for Saginae. Black nodes indicate UFBoot ≥ 95 and SH‐aLRT ≥ 80; grey nodes indicate either UFBoot ≥ 95 or SH‐aLRT ≥ 80.
**Figure S11:**. Phylogeny of the Tetrigonidae family inferred using a maximum likelihood approach based on 96 Ultra‐Conserved Elements and Unique Single‐Copy Orthologs. Black nodes indicate UFBoot ≥ 95 and SH‐aLRT ≥ 80; grey nodes indicate either UFBoot ≥ 95 or SH‐aLRT ≥ 80.
**Figure S12:**. Phylogeny of the Tetrigonidae family inferred using a maximum likelihood approach based on the COI barcode. Black nodes indicate UFBoot ≥ 95 and SH‐aLRT ≥ 80; grey nodes indicate either UFBoot ≥ 95 or SH‐aLRT ≥ 80.
**Figure S13:**. Phylogeny of the Rhaphidophoridae family inferred using a maximum likelihood approach based on 29 Ultra‐Conserved Elements and Unique Single‐Copy Orthologs. Trog. stands for Troglophilinae and Doli. for Dolichopodaini. Black nodes indicate UFBoot ≥ 95 and SH‐aLRT ≥ 80; grey nodes indicate either UFBoot ≥ 95 or SH‐aLRT ≥ 80.
**Figure S14:**. Phylogeny of the Rhaphidophoridae family inferred using a maximum likelihood approach based on the COI barcode. Trog. stands for Troglophilinae and Doli. for Dolichopodaini. Black nodes indicate UFBoot ≥ 95 and SH‐aLRT ≥ 80; grey nodes indicate either UFBoot ≥ 95 or SH‐aLRT ≥ 80.
**Figure S15:**. Phylogeny of the Gryllidae family inferred using a maximum likelihood approach based on 79 Ultra‐Conserved Elements and Unique Single‐Copy Orthologs. Gtal. stands for Gryllotalpini, Nemo. and N. for Nemobiini, Pter. and P. for Pteronemobiini, G. for Gryllomorphini, Modi. for Modicogryllini, Oeca. and O. for Oecanthidi. Black nodes indicate UFBoot ≥ 95 and SH‐aLRT ≥ 80; grey nodes indicate either UFBoot ≥ 95 or SH‐aLRT ≥ 80.
**Figure S16:** Phylogeny of the Gryllidae family inferred using a maximum likelihood approach based on the COI barcode. Gtal. stands for Gryllotalpini, Nemo. and N. for Nemobiini, Pter. and P. for Pteronemobiini, G. for Gryllomorphini, Modi. for Modicogryllini, Oeca. and O. for Oecanthidi. Black nodes indicate UFBoot ≥ 95 and SH‐aLRT ≥ 80; grey nodes indicate either UFBoot ≥ 95 or SH‐aLRT ≥ 80.
**Table S1:** Summary of mapping statistics across Orthoptera families for Ultra‐Conserved Elements (UCEs) and Unique Single Copy Orthologs (USCOs). Summary statistics were not included for families with fewer than four species. Reference genomes were either family‐specific or from closely related taxa. Some genomes (e.g., *M. thalassinum* , *G. longicercus*, *E. oculatus* ) are used across multiple families due to limited genomic resources.
**Table S2:** List of Orthoptera mitogenomes considered to design probes targeting mitogenes.
**Table S3:** Orthopteran species used for ribosomal DNA (rDNA) sequencing.

## Data Availability

Raw genomic data can be found under NCBI BioProjects PRJNA1224719. The pipelines including custom scripts have been made available in the Github repository https://github.com/icarrasquer/Multi‐locus_marker_Swiss_Orthoptera. Metadata and results summaries have been made available on Zenodo: https://doi.org/10.5281/zenodo.15772585
