# Supplementary material for: Multi‐Locus Nuclear Marker Assessment of Genetic Diversity in Swiss Orthoptera Unveils Conservation Status Limitations
Source: Mol Ecol. 2026 Jun 6;35(11):e70392. doi: 10.1111/mec.70392 (PMC13241918; doi:10.1111/mec.70392)
Supplement: Supplementary file 1 — Figure S1: Bioinformatic pipeline used to retrieve UltraConserved Elements (UCE), Unique Single Copy Orthologs (USCO), ribosomal DNA, mitogenome and COI barcode from raw data. For UCE and USCO, orthology inference was conducted on four reference genomes using (i) PHYLUCE, (ii) BUSCO v4, (iii) probe mapping and (iv) alignment distance thresholds. Ribosomal DNA and mitochondrial genomes were reconstructed using SPAdes, BLAST as well as RagTag and MitoFinder, respectively. COI barcodes were extracted via BLAST using BOLD database references. Figure S2: Distribution of genetic distances between captured genomic regions for each reference genome and the probes used to target the UltraConserved Elements (UCE) and Unique Single Copy Orthologs (USCO). The correct group corresponds to distances between the probes and loci previously identified as UCE or USCO through mapping or using PHYLUCE pipeline or BUSCO v4. The dotted line indicates the maximum distance accepted for a region to be assigned as a UCE or USCO. Figure S3: Capture success of the multi‐locus marker. A: Heatmap of UltraConserved Elements, Unique Single Copy Orthologs, ribosomal DNA, mitochondrial genes and COI barcode. B: Distribution of the number of UltraConserved Elements and Unique Single Copy Orthologs capture per sample according to their family. Numbers in the upper squares indicate the number of samples per category. Figure S4: Correlation between the mean % of polymorphic sites found in the UltraConserved Elements and Unique Single Copy Orthologs and the % of polymorphic sites found in the COI barcode (A), the mitogenome (B) and the ribosomal DNA (C). The blue line indicates linear regression. Figure S5: Correlation between the mean % of polymorphic sites found in the UltraConserved Elements and Unique Single Copy Orthologs per species and the area (km2) occupied by the samples. The blue line indicates linear regression. Figure S6: A. Schematic representation of the “equi‐read‐arity” process and its i [file MEC-35-e70392-s001.docx]

**Supporting Information for**

Multi-locus nuclear marker assessment of genetic diversity in Swiss Orthoptera unveils conservation status limitations

**“Equi-read-arity” protocol**

The mean DNA fragment size showed significant heterogeneity, ranging from 100 to 5000 bp. This variability is likely linked to differences in species size, sample age, and the variability in drying conditions commonly associated with extensive field studies involving numerous collectors, such as those conducted during Red List updates. We anticipated that the conserved nuclear regions would constitute a minor portion of the samples’ libraries compared to the mitochondrial and ribosomal DNA, which are both highly repeated in each cell of the organism. To enhance their homogeneous capture and sequencing —a concept we refer to as equi-read-arity— we performed a calibration capture by pooling 96 samples with 2 uL of library each (Figure S6A). The pools were captured by hybridization using a modified MYbaits protocol (Arbor Biosciences) with the biotinylated nuclear probes, followed by washing to eliminate non-captured sequences. We used an inverse-touchdown approach, starting with a first capture at 55 °C, whose product was then captured a second time at 65 °C to improve the stringency of the reaction and increase the coverage of rare targets. The enriched libraries were pooled by pipetting 2 uL and sequenced on a lane of an Illumina MiSeq with 150-bp reads.

Following demultiplexing with Cutadapt v2.0 [1], the number of reads corresponding to UltraConserved Elements (UCE) and Unique Single Copy Orthologs (USCO) was quantified for each sample by assembling the reads using SPAdes [2] and aligning them against the UCE and USCO probes using BLAST [3]. Samples were then categorized into groups based on their read concentration (number of reads per µL), and new pools were prepared accordingly (Figure S6B).

We performed two separate hybridization captures using the libraries pool: one with the probes targeting the conserved UCE and USCO, nuclear regions and the other with the combination of mitochondrial and ribosomal probes. The product of both captures was pooled at a ratio of 9:1, for UCE + USCO and mitogenome + ribosomal DNA respectively, and libraries were sequenced on Illumina NextSeq with 150-bp reads.

**Capture efficiency**

The number of captured loci per sample ranged from 1 to 61 per sample in Tettigoniidae (mean length 294 bp, sd = 185), with 27 loci passing all filters for phylogenetic inference (PI). Rhaphidophoridae showed a narrower range, with 9 to 18 loci captured (mean length 220 bp, sd = 140), and 29 loci used for PI. Gryllidae samples had 67 to 226 loci recovered (mean length 310 bp, sd = 205), resulting in 79 loci suitable for PI. For Acrididae, 75 to 305 loci per sample were recovered (mean length 294 bp, sd = 178), with 260 appropriate for PI. Tetrigidae samples yielded between 24 and 184 loci (mean length 235 bp, sd = 106), with 96 loci used for PI. The ribosomal DNA was successfully captured for 91.9% of samples, the COI barcode for 71% of samples, while success rates for individual mitochondrial genes ranged from 63.7% for the entire COI gene to only 3.3% for ATP8. Unlike UCE and USCO loci, we did not observe a family-dependent effect on capture success. Regarding the museum specimens incorporated in the sampling of each of the four families, we successfully captured up to 236 UCE and USCO loci, with an average of 68 loci per specimen (mean length 173 bp, sd = 14, Figure S3).

The heterogeneity in capture between samples is linked to the heterogeneous quality of the extracted DNA and the phylogenetic distribution of the reference genomes used to first define the UCE and USCO probes. The case is exemplified in Tettigoniidae family, for which we had no reference genome at the time this study began, and which is therefore associated with a more limited number of loci, i.e. 27 nuclear loci. Moreover, we suspect among-samples competition during capture in relation to their phylogenetic proximity to the probes. In the future, this could be resolved by grouping samples from the same clade during capture. These limitations highlight the importance of high-quality genomes for designing appropriate markers, developing clade-specific UCE datasets and improving their scalability [4]. The long divergence time of the orthopteran order and the high levels of gene paralogy found in their genomes, combined with the use of a mapping-based pipeline rather than an assembly-based one, may explain the exonic nature of a large fraction of the retrieved UCE.

**Phylogenetic inference - results**

With respect to the relative performance of the different markers, the multi-locus marker comprising UCE and USCO loci outperformed the ribosomal DNA, the mitogenome, and the COI barcode, demonstrating the highest phylogenetic resolution. Regarding the mitogenome, the inclusion of additional mitochondrial genes alongside the COI barcode did not increase the number of monophyletic species identified. It failed to retrieve as monophyletic species that the COI barcode had identified as such, including *Nemobius sylvestris*, *Conocephalus fuscus*, and *Tetrix subulata*. In contrast, rDNA sequences improved the monophyletic species yield compared to COI barcode or mitochondrial genes, as for example in species from genera *Omocestus* or *Stenobothrus*. However, polytomies frequently occurred at the intraspecific level, along with overall short branches and low node support, revealing low informativeness of the ribosomal marker. This is exemplified by e.g., i) the three *Ephippiger* species—*E. persicarius*, *E. diurnus diurnus*, and *E. terrestri bormansi*— retrieved as paraphyletic or ii) the poorly resolved phylogeny (also exhibiting very short branches) obtained for the Tetrigidae family. Given the limitations of the complete mitogenome and ribosomal DNA, we focused further stages of this study on the comparison between phylogenies obtained from the COI barcode, which has been widely used for species identification, and those generated using the set of UCE and USCO loci developed here.

Concerning the phylogenetic reconstruction of Acrididae, the trees inferred using the multi-locus marker and the COI barcode phylogeny differed significantly (Figures S7 and S8). For instance, in the Stenobothrini tribe, the three *Omocestus* species (*O. haemorrhoidalis, O. rufipes, O. viridulus*) and the four *Stenobothrus* species (*S. nigromaculatus, S. lineatus, S. rubicundulus, S. stigmaticus*) found in Switzerland are each retrieved as monophyletic, whereas the COI barcode fails to differentiate them (Figure 2). At the genus level, the COI barcode retrieves the *Stenobothrus* as paraphyletic, placing *Stenobothrus* *stigmaticus* in a sister clade alongside *Myrmeleotettix maculatus*. In contrast, using the multi-locus marker, the whole genus *Stenobothrus* is found to be monophyletic, with *M. maculatus* correctly positioned as a sister group to the genus. Both *M. maculatus* and *Chorthippus pullus* belong to the Gromphocerini tribe, yet they are nested within the Stenobothrini clade in phylogenies inferred using both nuclear loci and COI barcode. Concerning the Gomphocerini tribe, the placement of *C. apricarius* diverges between COI barcode, which finds *C. apricarius* within the *Chorthippus biguttulus* complex, and the multi-locus marker, which groups *C. apricarius* with *Gomphocerus sibiricus* and *Staurodeus scalaris*. Additionally, *Gomphocerippus rufus, Gomphocerus sibiricus*, and *Staurodeus scalaris* all fall within the *Chorthippus* clade in both COI barcode and UCE and USCO reconstructions; however, only the nuclear loci indicate that these species form monophyletic clades. The *Chorthippus biguttulus* complex, including *C. biguttulus, C. brunneus, C. eisentrauti, C. mollis* and *C. apricarius*, as well as the sister species *Pseudochorthippus montanus* and *P. parallelus* remain unresolved using either COI barcode or UCE and USCO markers. Within the Melanoplini tribe, the sister species *Miramella alpina* and *Nadigella formosanta* do not form separate clades, as *N. formosanta* individuals are nested within the *M. alpina* clade. Additionally, *M. alpina* is divided in two clades using UCE and USCO, with one clade containing individuals from the Grisons canton (located in the Eastern part of Switzerland). Such structure is not observed when using COI barcode sequences.

For the Tettigoniidae family (Figures S9 and S10), both COI and multi-locus marker phylogenies recovered the genus *Leptophyes*, the Platycleidini tribe and the sister species *Roeseliana roeselii* and *Roeseliana azami minor* as paraphyletic, while supporting the monophyly of the *Metrioptera* genus—despite a recent taxonomic trend to separate certain species into distinct genera (e.g., *Roeseliana roeseli*i and *Bicolorana bicolor*). In contrast to the COI barcode, the UCE and USCO loci supported the monophyly of the genus *Antaxius* but were ineffective in resolving species within genera *Tettigonia* and *Meconema*. Unfortunately, due to an insufficient number of samples retrieved for *Platycleis albopunctata grisea*, we were unable to assess the genetic structure between the subspecies *Platycleis albopunctata albopunctata* and *P. a. grisea*.

For the Tetrigidae, Rhaphidophoridae, and Gryllidae families, all species were retrieved as monophyletic with both types of markers (Figures S11 to S16). Furthermore, the two subspecies of *Tetrix bipunctata*, ssp. *bipunctata* and ssp. *kraussi* did not form separate clades. At the interspecific level, the COI barcode and UCE + USCO loci exhibited different topologies, indicating mitochondrial-nuclear discordance. In the case of the Gryllidae, where the number of species is reduced, nearly every species represents a different tribe. Therefore, the observed mitochondrial-nuclear discordances might reflect distinct evolutionary histories during the early steps of lineage divergence, particularly illustrated by the placement of *Nemobius sylvestris* (Nemobiini) and *Oecanthus pellucens* (Oecanthidi).

It should be noted that numts (revealed by the presence of STOP codons along the sequence) can still be found in the COI barcode phylogenies, despite the alignment cleaning steps. Examples include ORT-586 (*Chorthippus pullus*), ORT-542 (*Omocestus viridulus*), ORT-547 (*Omocestus haemorrhoidalis*), ORT-141 (*Chorthippus dorsatus*), ORT-088 (*Pholidoptera griseoaptera*), and ORT-557 (*Gryllus campestris*).

**Phylogenetic inference - discussion**

With regard to the phylogenetic reconstruction of Acrididae, our results with UCE and USCO markers highlight the increase in phylogenetic resolution, especially in the case of the rapid radiation of Gomphocerinae. This improvement is illustrated by the observed monophyly of species within genera *Omocestus* and *Stenobothrus*. In addition, UCE and USCO analyses support the classification of *Pseudochorthippus* *parallelus* and *P. montanus* into the genus *Pseudochorthippus*, consistent with the work of Schmidt et al. [5]. Although the COI barcode similarly supports the genus *Pseudochorthippus*, it places instead the latter within the Stenobothrini clade. We support the placement of *Gomphocerippus rufus*, *Gomphocerus sibiricus* and *Stauroderus scalaris* within the *Chorthippus* clade, consistent with findings by Hawlitschek and colleagues [6] using a dataset of 540 nuclear transcriptomes. Concerning the paraphyly of *P. montanus* and *P. parallelus*, it is important to note that the two individuals of *P. montanus*, ORT-119 and ORT-469, placed within the *P. parallelus* clade are suspected to be hybrids. This suspicion is based on the known hybridization between the species [7] and the intermediate characteristics of the specimens, particularly in morphological traits such as the relative placement of the stigma within the elytra. However, our phylogenetic reconstruction using UCE and USCO markers failed to retrieve as monophyletic the *Chorthippus biguttulus* complex species as shown by Schmidt et al. [8] using ddRAD data or by Nolen et al. [9] using complete transcriptomes. The latter reported high levels of incomplete lineage sorting (ILS) within the group in both mitochondrial and nuclear genes. This was attributed to recent radiation during the mid-Pleistocene, followed by gene flow, which resulted in strong introgression throughout the genome and low genetic distinctiveness. Such patterns of ILS and introgression are consistent with the paralogy observed in our phylogeny. Additionally, two members of the Gomphocerini tribe, *Chorthippus pullus* and *Myrmyleotettix maculatus*, are unexpectedly nested within the Stenobothrini clade. In Podismini, the genus *Podisma* is identified as an outgroup to *Miramella* and *Odontopodisma*, consistent with previous findings [9]. Further analyses are required to clarify whether *Miramella alpina* should be classified as distinct species or subspecies and its relation to *Nadigella formosanta*. These findings, together with previous studies [6], highlight the need for a comprehensive revision of the Acrididae taxonomy.

For Tetrigidae, phylogenetic reconstructions using mitochondrial and ribosomal genes have been conducted for various species within the family [10, 11]. However, only the COI barcode phylogeny from Hawlitschek et al. [12] was previously available for species occurring in Switzerland. Our results do not support the distinction of *Tetrix bipunctata* and *Tetrix kraussi*. These two species, also considered as subspecies, are theoretically distinguished by a single morphological trait and occurred in sympatry, with microhabitat differences observed in a single location in Germany [13].

Despite extensive genomic studies on Acrididae, research on Tettigoniidae (katydids) primarily relies on ribosomal DNA and mitochondrial genes [14, 15, 16], with limited broader genomic studies available that typically do include few species occurring in Switzerland [17, 18]. The absence of a Tettigoniidae genome during the development of the multi-locus marker used here significantly impacted the capture success for samples from this family, resulting in fewer loci passing the quality filters compared to other groups, such as Acrididae. However, the 27 loci retrieved were informative enough to infer a well-supported and resolutive phylogeny. The multi-locus marker phylogeny aligns with the current taxonomy and supports the monophyly of genera *Antaxius* and *Metrioptera*, as well as of most other species, genera, and tribes, with exceptions for *Tettigonia*, *Leptophyes*, and *Roeseliana roeselii*. While both marker types identified the same number of species, the lower resolution of UCE and USCO to identify *Meconema* or *Tettigonia* compared to the COI may be due to the shorter coalescence time of mitochondrial DNA compared to nuclear DNA [19, 20]. Consistent with Mugleston et al. [16], we also recovered the tribe Platycleidini as paraphyletic.

The most recent phylogenetic analyses of Gryllidae, based on complete mitogenomes [21] or seven genetic markers—three mitochondrial and four nuclear [22]—support the same relationships among tribes, placing Nemobiinae as an outgroup to Gryllini and Oecanthini. The family Gryllotalpidae is also consistently recovered as an outgroup to Gryllidae, aligning with previous findings [22,23].

For Rhaphidophoridae, the monophyly of *Troglophilus* is well-supported, corroborating previous results [24, 25].

The above mentioned results have several taxonomic implications that will need to be addressed. The genus *Chorthippus* is paraphyletic, with species distributed across different clades and intermixed with *S. scalaris*, *G. rufus*, and *G. sibiricus*. The placement of *C. pullus* requires the description of a new genus. The placement of *M. maculatus* and *C. pullus* supports their reclassification from the tribe Gomphocerini to Stenobothrini. The *Tetrix kraussi* form lack phylogenetic support and should no longer be considered. The tribe Platycleidini also lacks phylogenetic support and warrants further investigation. Furthermore, several parts of the Orthoptera tree may need further investigation to clarify phylogenetic relationships, including between genera *Miramella* and *Nadigella*, addressing the species delimitation within the genus *Podismopsis* across Eurasia and investigating genera definitions within Barbistini.

**Codon placement of variants**

Although most of the UCE and USCO captured sequences were exons, they exhibited within-individual variability, with a mean of 0.966 SNP per loci and sample. To further explore SNP placement within UCE and USCO and their evolutionary significance, we examined whether these exons were protein-coding and determined their positions within the codons using the VariantAnnotation package (Obenchain et al. 2014) from Bioconductor. Thirty-nine percents of these SNP were synonymous mutations, while 57% were non-synonymous and 4% were nonsense mutations.

**Figures and Tables**

**Figure S1.** Bioinformatic pipeline used to retrieve UltraConserved Elements (UCE), Unique Single Copy Orthologs (USCO), ribosomal DNA, mitogenome and COI barcode from raw data. For UCE and USCO, orthology inference was conducted on four reference genomes using (i) PHYLUCE, (ii) BUSCO v4, (iii) probe mapping and (iv) alignment distance thresholds. Ribosomal DNA and mitochondrial genomes were reconstructed using SPAdes, BLAST as well as RagTag and MitoFinder, respectively. COI barcodes were extracted via BLAST using BOLD database references.

**Figure S2.** Distribution of genetic distances between captured genomic regions for each reference genome and the probes used to target the UltraConserved Elements (UCE) and Unique Single Copy Orthologs (USCO). The correct group corresponds to distances between the probes and loci previously identified as UCE or USCO through mapping or using PHYLUCE pipeline or BUSCO v4. The dotted line indicates the maximum distance accepted for a region to be assigned as a UCE or USCO.

**Figure S3.** Capture success of the multi-locus marker. A: Heatmap of UltraConserved Elements, Unique Single Copy Orthologs, ribosomal DNA, mitochondrial genes and COI barcode. B: Distribution of the number of UltraConserved Elements and Unique Single Copy Orthologs capture per sample according to their family. Numbers in the upper squares indicate the number of samples per category.

**Figure S4.** Correlation between the mean % of polymorphic sites found in the UltraConserved Elements and Unique Single Copy Orthologs and the % of polymorphic sites found in the COI barcode (A), the mitogenome (B) and the ribosomal DNA (C). The blue line indicates linear regression.

**Figure S5.** Correlation between the mean % of polymorphic sites found in the UltraConserved Elements and Unique Single Copy Orthologs per species and the area (km2) occupied by the samples. The blue line indicates linear regression.

**Figure S6.** A. Schematic representation of the “equi-read-arity” process and its integration into final capture. B. Coverage plots and pooling categories.

**Figure S7**. Phylogeny of the Acrididae family inferred using a maximum likelihood approach based on 260 Ultra-Conserved Elements and Unique Single-Copy Orthologs. Arcy. stands for Arcypterini, C. for Cyrtacanthacridini Chry. for Chrysochraontini, Epac. for Epacromiini, Gomp. for Gomphocerini, Locu. for Locustini, P. for Pezotettiginae and Sphi. for Sphingonotini. Black nodes indicate UFBoot ≥ 95 and SH-aLRT ≥ 80; grey nodes indicate either UFBoot ≥ 95 or SH-aLRT ≥ 80.

.

**Figure S8**. Phylogeny of the Acrididae family inferred using a maximum likelihood approach based on the COI barcode. A. stands for Arcypterini, B. for Bryodemini, C. for Cyrtacanthacridini, Call. for Calliptamini, E. for Epacromiini, G. and Gomp. for Gomphocerini, L. for Locustini, P. for Pezotettiginae, Para. for Parapleurini, Podi. for Podismini, S. and Sten. for Stenobothrini and Sphi. for Sphingonotini. Black nodes indicate UFBoot ≥ 95 and SH-aLRT ≥ 80; grey nodes indicate either UFBoot ≥ 95 or SH-aLRT ≥ 80.

**Figure S9**. Phylogeny of the Tettigoniidae family inferred using a maximum likelihood approach based on 27 Ultra-Conserved Elements and Unique Single-Copy Orthologs. Barb. and B. stand for Barbitistini, Copi. for Copiphorini, Cono. for Conocephalini, Ephi. for Ephippigerini, M. for Meconematini, P. for Platycleidini, Phan. for Phaneropterini and S. for Saginae. Black nodes indicate UFBoot ≥ 95 and SH-aLRT ≥ 80; grey nodes indicate either UFBoot ≥ 95 or SH-aLRT ≥ 80.

**Figure S10**. Phylogeny of the Tettigoniidae family inferred using a maximum likelihood approach based on the COI barcode. Copi. stands for Copiphorini, Cono. for Conocephalini, Ephi. for Ephippigerini, Meco. for Meconematini, P. for Platycleidini, Phan. for Phaneropterini and S. for Saginae. Black nodes indicate UFBoot ≥ 95 and SH-aLRT ≥ 80; grey nodes indicate either UFBoot ≥ 95 or SH-aLRT ≥ 80.

**Figure S11**. Phylogeny of the Tetrigonidae family inferred using a maximum likelihood approach based on 96 Ultra-Conserved Elements and Unique Single-Copy Orthologs. Black nodes indicate UFBoot ≥ 95 and SH-aLRT ≥ 80; grey nodes indicate either UFBoot ≥ 95 or SH-aLRT ≥ 80.

**Figure S12**. Phylogeny of the Tetrigonidae family inferred using a maximum likelihood approach based on the COI barcode. Black nodes indicate UFBoot ≥ 95 and SH-aLRT ≥ 80; grey nodes indicate either UFBoot ≥ 95 or SH-aLRT ≥ 80.

**Figure S13**. Phylogeny of the Rhaphidophoridae family inferred using a maximum likelihood approach based on 29 Ultra-Conserved Elements and Unique Single-Copy Orthologs. Trog. stands for Troglophilinae and Doli. for Dolichopodaini. Black nodes indicate UFBoot ≥ 95 and SH-aLRT ≥ 80; grey nodes indicate either UFBoot ≥ 95 or SH-aLRT ≥ 80.

**Figure S14**. Phylogeny of the Rhaphidophoridae family inferred using a maximum likelihood approach based on the COI barcode. Trog. stands for Troglophilinae and Doli. for Dolichopodaini. Black nodes indicate UFBoot ≥ 95 and SH-aLRT ≥ 80; grey nodes indicate either UFBoot ≥ 95 or SH-aLRT ≥ 80.

**Figure S15**. Phylogeny of the Gryllidae family inferred using a maximum likelihood approach based on 79 Ultra-Conserved Elements and Unique Single-Copy Orthologs. Gtal. stands for Gryllotalpini, Nemo. and N. for Nemobiini, Pter. and P. for Pteronemobiini, G. for Gryllomorphini, Modi. for Modicogryllini, Oeca. and O. for Oecanthidi. Black nodes indicate UFBoot ≥ 95 and SH-aLRT ≥ 80; grey nodes indicate either UFBoot ≥ 95 or SH-aLRT ≥ 80.

**Figure S16.** Phylogeny of the Gryllidae family inferred using a maximum likelihood approach based on the COI barcode. Gtal. stands for Gryllotalpini, Nemo. and N. for Nemobiini, Pter. and P. for Pteronemobiini, G. for Gryllomorphini, Modi. for Modicogryllini, Oeca. and O. for Oecanthidi. Black nodes indicate UFBoot ≥ 95 and SH-aLRT ≥ 80; grey nodes indicate either UFBoot ≥ 95 or SH-aLRT ≥ 80.

| Samples family | Reference genome | GenBank accession number | % reads mapped | % reads mapped after cleaning | Loci length for phylogenetic inference |
| --- | --- | --- | --- | --- | --- |
| Tettigoniidae | *Meconema thalassinum* | GCA_946902985.2 | 81.9% ± 10.6 | 52.1% ± 23.4 | 150.61 |
| Rhaphidophoridae | *Meconema thalassinum* | GCA_946902985.2 | 70.1% ± 3.18 | 36.7% ± 3.35 | 149.66 |
| Gryllidae | *Gryllus longicercus* | GCA_038098605.1 | 81.0% ± 17.4 | 61.5% ± 22.1 | 191.79 |
| Gryllotalpidae | *Gryllus longicercus* | GCA_038098605.1 | - | - | - |
| Acrididae | *Schistocerca gregaria* | GCF_023897955.1 | 93.9% ± 5.93 | 72.1% ± 12.9 | 191.67 |
| Tetrigidae | *Eucriotettix oculatus* | GCA_034510155.1 | 81.4% ± 21.0 | 63.6% ± 23.4 | 192.56 |
| Tridactylidae | *Eucriotettix oculatus* | GCA_034510155.1 | - | - | - |

**Table S1.** Summary of mapping statistics across Orthoptera families for Ultra‑Conserved Elements (UCEs) and Unique Single Copy Orthologs (USCOs). Summary statistics were not included for families with fewer than four species. Reference genomes were either family-specific or from closely related taxa. Some genomes (e.g., *M. thalassinum*, *G. longicercus*, *E. oculatus*) are used across multiple families due to limited genomic resources.

**Table S2.** List of Orthoptera mitogenomes considered to design probes targeting mitogenes.

| Family | Species | GenBank accession number |
| --- | --- | --- |
| Gryllotapidae | *Gryllotalpa orientalis* | NC_006678.1 |
| Rhaphidophoridae | *Troglophilus neglectus* | NC_011306.1 |
| Gryllidae | *Oecanthus sinensis* | NC_034799.1 |
| Tettigoniidae | *Phaneroptera nigroantennata* | NC_034757.1 |
| Acrididae | *Oedaleus decorus* | NC_011115.1 |
| Acrididae | *Calliptamus italicus* | NC_011305.1 |
| Tetrigidae | *Tetrix japonica* | NC_018543.1 |
| Tridactylidae | *Ellipes minuta* | NC_014488 |

**Table S3.** Orthopteran species used for ribosomal DNA (rDNA) sequencing.

| Family | Species | GenBank accession number |
| --- | --- | --- |
| Acrididae | *Podismopsis keisti* | PV867681.1 |
| Acrididae | *Aoilopus strepens* | PV867682.1 |
| Acrididae | *Epacromius tergestinus* | PV867684.1 |
| Acrididae | *Oedaleus decorus* | PV867688.1 |
| Gryllidae | *Pteronemobius lineolatus* | PV867685.1 |
| Gryllidae | *Gryllus campestris* | PV867686.1 |
| Rhaphidophoridae | *Troglophilus cavicola* | PV867683.1 |
| Tettigoniidae | *Leptophyes albovittata* | SUB15409869 |
| Tettigoniidae | *Conocephalus fuscus* | SUB15409869 |
| Tettigoniidae | *Meconema thalassinum* | PV867687.1 |
| Tettigoniidae | *Anonconotus alpinus* | PV867689.1 |

**SI References**

1. Martin, M. (2011). Cutadapt removes adapter sequences from high-throughput sequencing reads. *EMBnet.journal*, 17, 10–12.

2. Bankevich, A., Nurk, S., Antipov, D., Gurevich, A.A., Dvorkin, M., Kulikov, A.S., *et al.* (2012). SPAdes: a new genome assembly algorithm and its applications to single-cell sequencing. *Journal of Computational Biology*, 19, 455–477.

3. Camacho, C., Coulouris, G., Avagyan, V., Ma, N., Papadopoulos, J., Bealer, K., *et al.* (2009). BLAST+: architecture and applications. *BMC Bioinformatics*, 10, 421.

4. Gustafson, G.T., Glynn, R.D., Short, A.E.Z., Tarasov, S. & Gunter, N.L. (2023). To design, or not to design? Comparison of beetle ultraconserved element probe set utility based on phylogenetic distance, breadth, and method of probe design. *Insect Systematics and Diversity*, 7, 4.

5. Schmidt, R., Dufresnes, C., Krištín, A., Künzel, S., Vences, M. & Hawlitschek, O. (2024). Phylogenetic insights into Central European Chorthippus and Pseudochorthippus (Orthoptera: Acrididae) species using ddRADseq data. *Molecular Phylogenetics and Evolution*, 193, 108012.

6. Hawlitschek, O., Ortiz, E.M., Noori, S., Webster, K.C., Husemann, M. & Pereira, R.J. (2022). Transcriptomic data reveals nuclear-mitochondrial discordance in Gomphocerinae grasshoppers (Insecta: Orthoptera: Acrididae). *Molecular Phylogenetics and Evolution*, 170, 107439.

7. Hochkirch, A. & Lemke, I. (2011). Asymmetric mate choice, hybridization, and hybrid fitness in two sympatric grasshopper species. *Behavioral Ecology and Sociobiology*, 65, 1637–1645.

8. Nolen, Z.J., Yildirim, B., Irisarri, I., Liu, S., Groot Crego, C., Amby, D.B., *et al.* (2020). Historical isolation facilitates species radiation by sexual selection: Insights from Chorthippus grasshoppers. *Molecular Ecology*, 29, 4985–5002.

9. Chintauan-Marquier, I.C., Amédégnato, C., Nichols, R.A., Pompanon, F., Grandcolas, P. & Desutter-Grandcolas, L. (2014). Inside the Melanoplinae: new molecular evidence for the evolutionary history of the Eurasian Podismini (Orthoptera: Acrididae). *Molecular Phylogenetics and Evolution*, 71, 224–233.

10. Chen, Y.-Z., Deng, W.-A., Wang, J.-M., Lin, L.-L. & Zhou, S.-Y. (2018). Phylogenetic relationships of Scelimeninae genera (Orthoptera: Tetrigoidea) based on COI, 16S rRNA and 18S rRNA gene sequences. *Zootaxa*, 4482, 392–400.

11. Li, R., Ying, X., Deng, W., Rong, W. & Li, X. (2021). Mitochondrial genomes of eight Scelimeninae species (Orthoptera) and their phylogenetic implications within Tetrigoidea. *PeerJ*, 9, e10523.

12. Hawlitschek, O., Morinière, J., Lehmann, G.U.C., Lehmann, A.W., Kropf, M., Dunz, A., *et al.* (2017). DNA barcoding of crickets, katydids and grasshoppers (Orthoptera) from Central Europe with focus on Austria, Germany and Switzerland. *Molecular Ecology Resources*, 17, 1037–1053.

13. Moser, V., Baur, H., Lehmann, A.W. & Lehmann, G.U.C. (2021). Two species? - Limits of the species concepts in the pygmy grasshoppers of the Tetrix bipunctata complex (Orthoptera, Tetrigidae). *Zookeys*, 1043, 33–59.

14. Çıplak, B., Yahyaoğlu, Ö. & Uluar, O. (2021). Revisiting Pholidopterini (Orthoptera, Tettigoniidae): Rapid radiation causes homoplasy and phylogenetic instability. *Zoologica Scripta*, 50, 225–240.

15. Mugleston, J.D., Song, H. & Whiting, M.F. (2013). A century of paraphyly: a molecular phylogeny of katydids (Orthoptera: Tettigoniidae) supports multiple origins of leaf-like wings. *Molecular Phylogenetics and Evolution*, 69, 1120–1134.

16. Mugleston, J.D., Naegle, M., Song, H. & Whiting, M.F. (2018). A comprehensive phylogeny of Tettigoniidae (Orthoptera: Ensifera) reveals extensive ecomorph convergence and widespread taxonomic incongruence. *Insect Systematics and Diversity*, 2, 5.

17. Shin, S., Baker, A.J., Enk, J., McKenna, D.D., Foquet, B., Vandergast, A.G., *et al.* (2024). Orthoptera-specific target enrichment (OR-TE) probes resolve relationships over broad phylogenetic scales. *Scientific Reports*, 14, 21377.

18. Song, H., Béthoux, O., Shin, S., Donath, A., Letsch, H., Liu, S., et al. (2020). Phylogenomic analysis sheds light on the evolutionary pathways towards acoustic communication in Orthoptera. *Nature Communications*, 11, 4939.

19. Maddison, W.P. & Knowles, L.L. (2006). Inferring phylogeny despite incomplete lineage sorting. *Systematic Biology*, 55, 21–30.

20. Palumbi, S.R., Cipriano, F. & Hare, M.P. (2001). Predicting nuclear gene coalescence from mitochondrial data: the three-times rule. *Evolution*, 55, 859–868.

21. Dong, J., Liu, Y., Tan, M.K., Wahab, R.A., Nattier, R., Chifflet-Belle, P., *et al.* (2024). Museomics allows comparative analyses of mitochondrial genomes in the family Gryllidae (Insecta, Orthoptera) and confirms its phylogenetic relationships. *PeerJ*, 12, e17734.

22. Chintauan-Marquier, I.C., Legendre, F., Hugel, S., Robillard, T., Grandcolas, P., Nel, A., *et al.* (2016). Laying the foundations of evolutionary and systematic studies in crickets (Insecta, Orthoptera): a multilocus phylogenetic analysis. *Cladistics*, 32, 54–81.

23. Ma, Y. & Miao, Y. (2022). Mitogenomic comparison of the mole crickets Gryllotalpidae with the phylogenetic implications (Orthoptera: Ensifera). *Insects*, 13, 919.

24. Beasley-Hall, P.G., Trewick, S.A., Eberhard, S.M., Zwick, A., Reed, E.H., Cooper, S.J.B., *et al.* (2024). Molecular phylogenetics illuminates evolutionary history and hidden diversity of Australian cave crickets (Orthoptera: Rhaphidophoridae). *bioRxiv,* 2024-08.

25. Kim, D.-Y., Kim, S., Song, H. & Shin, S. (2024). Phylogeny and biogeography of the wingless orthopteran family Rhaphidophoridae. *Communications Biology*, 7, 401.
